# Early Onset of Laying and Bumblefoot Favor Keel Bone Fractures

**DOI:** 10.3390/ani5040406

**Published:** 2015-11-27

**Authors:** Sabine G. Gebhardt-Henrich, Ernst K. F. Fröhlich

**Affiliations:** 1 Center for Proper Housing: Poultry and Rabbits, Division of Animal Welfare, University of Bern, Burgerweg 22, Zollikofen CH-3052, Switzerland; 2 Center for Proper Housing: Poultry and Rabbits, Federal Veterinary Office, Burgerweg 22, Zollikofen CH-3052, Switzerland; E-Mail: ekf.froehlich@bluewin.ch

**Keywords:** laying hen, keel bone fracture, laying rate, behavior, welfare

## Abstract

**Simple Summary:**

Numerous studies have documented a high prevalence of keel bone fractures in laying hens. In this longitudinal study, 80 white and brown laying hens were regularly checked for keel bone deviations and fractures while egg production was individually monitored. About 62% of the hens had broken keel bones at depopulation. More new fractures occurred during the time when laying rates were highest. Hens with broken keel bones at depopulation had laid their first egg earlier than hens with intact keel bones. All birds with bumblefoot on both feet had a fracture at depopulation.

**Abstract:**

Numerous studies have demonstrated influences of hybrid, feed, and housing on prevalence of keel bone fractures, but influences of behavior and production on an individual level are less known. In this longitudinal study, 80 white and brown laying hens were regularly checked for keel bone deviations and fractures while egg production was individually monitored using Radio Frequency Identification (RFID) from production until depopulation at 65 weeks of age. These focal birds were kept in eight pens with 20 hens per pen in total. About 62% of the hens had broken keel bones at depopulation. The occurrence of new fractures was temporally linked to egg laying: more new fractures occurred during the time when laying rates were highest. Hens with fractured keel bones at depopulation had laid their first egg earlier than hens with intact keel bones. However, the total number of eggs was neither correlated with the onset of egg laying nor with keel bone fractures. All birds with bumblefoot on both feet had a fracture at depopulation. Hens stayed in the nest for a longer time during egg laying during the ten days after the fracture than during the ten days before the fracture. In conclusion, a relationship between laying rates and keel bone fractures seems likely.

## 1. Introduction

Keel bone damage including deviations of the bone and fractures are common in laying hens, especially in non-cage housing systems, and present a major risk to the well-being of the animals [1,2,3,4]. The presence of perchable objects (e.g., dedicated perches, as well as other items like water pipes) and their height are thought to be a primary factor in causing keel bone fractures due to collisions [5]. Alternatively, fractures of the keel bone could also be caused by the pressure perches exert on the perching bird [6,7,8]. Apart from perches and other housing equipment, de-mineralized bone, due to a high egg laying rate could influence the prevalence of keel bone fractures. Fleming *et al.* [9] concluded that the most important factor influencing bone strength was genetics followed by the environment and particularly nutrition. Disentangling these factors is difficult. Flocks can differ substantially in the prevalence of keel bone fractures that could not be explained by known variables (hybrid, flock size, aviary type, free-range) [1]. In order to determine links between production and the presence of keel bone fractures, hens in this study were individually monitored throughout production for egg laying, occurrence of keel bone deviations and fractures, and behavior.

Fractures in birds might be similarly painful, as in humans, and do appear to affect hens in a variety of ways [10,11,12,13]. In comparison to hens without fractures, hens with fractures took more time to fly up or down to a perch, were slower to complete a course with obstacles [11], and visited the open range less [14]. Production traits differed in hens with and without fractures, as hens with fractures laid eggs with thinner eggs shells [11]. However, in these cases, hens were compared after the fractures had occurred and, therefore, it is unclear whether the hens already differed in the investigated traits before fracture occurred.

In the present longitudinal study, white and brown laying hens were palpated regularly between 18 and 65 weeks of age and their rate of egg laying and behavior was monitored at the individual level. We hypothesized that hens with a higher egg production were more likely to incur fractures. We also wanted to know if hens changed their behavior after fracturing their keel bone and whether this behavioral change could be used to determine when birds had fractures.

## 2. Experimental Section

### 2.1. Animals and Housing

The experiment was approved by the Veterinary Office of the Canton of Bern to adhere to the Swiss regulations about experiments using animals. Eighty white (Nick Chick) and 80 brown laying hens (Brown Nick) [15] were obtained as one-day-old chicks and raised in a rearing aviary system Inauen Natura (R. Inauen AG, Appenzell, Switzerland), which was equipped with perches, automatic chain-feeders, nipple drinkers, heaters, humidifier and manure belt. At 18 weeks, they were assigned to eight pens with ten white and ten brown hens each. The pens (3 × 4 × 2 m) were separated by solid barriers and were equipped with four single nests with a plastic platform along the entrance, six perches in a pyramid configuration, feeder, drinker, litter (wood shavings), and a manipulable building block as enrichment. Perches were made of steel covered by a thin layer of plastic and had a diameter of 37 mm (Sanatherm, R. Inauen AG, Appenzell, Switzerland). The perches were 58, 112, and 168 cm above the litter. The horizontal distance between the lowest, middle, and highest perches was 122, 76, and 30 cm, respectively. The platform in front of the nests was 70 cm high. The wooden nests were 26 cm wide and 30 cm deep with a cut red curtain at the front to accommodate a single hen at a time. Water and a standard laying hen diet were provided *ad libitum*. Natural daylight was excluded and artificial day length was successively raised from 10 to 15 h over seven weeks (week 1: 90 min., week 2: 60 min., week 3 and following 30 min.). At transfer to the pens, hens were fitted with Radio Frequency Identification (RFID) tags (ø 4.0/34.0 mm Hitag S 2048 bits, 125 kHz) which were put into wing tags and fastened to the legs with bands (both products from Roxan, Scotland). Half of the hens were additionally color marked on the neck or tail [16]. The color was refreshed as needed. The flock was depopulated at 65 weeks of age. 

### 2.2. Data Collection

All eggs in the nests and on the floor were counted daily approximately 12 h after lights came on. The birds’ duration in the nest was recorded by the Gantner Pigeon System which is a 125 kHz RFID registration system (6780 Schruns, Austria [17]) from 19 until 64 weeks of age. The floor of the nest consisted of a sloped RFID antenna (PLB 765, Gantner Pigeon System, Schruns, Austria). When eggs were laid, a light beam shining across the back of the nest was broken and triggered an entry into a datafile containing the number of the nest and the time (Eilog, Schaer-Consulting, Bern, Switzerland). This was matched with the registration of a hen in the respective nest by the RFID system and thus the egg was linked to a particular hen. The eighty color marked hens were palpated 43 times by the same observer. At the time of palpation, the observer was “blind” to the previous score of the particular animal at the previous palpation measurement. During the dark phase, the hen was held and the keel bone was palpated by running two fingers down the edge to feel for alterations like s-shaped deviations, bumps or depressions. The following scoring system was used: 4 = normal keel bone, 3 = slight deviation, 2 = moderate fracture, 1 = severe fracture. The terminology of deviations and fractures follows Casey-Trott *et al.* [18]. Scoring had been calibrated by S. Käppeli [19,20]. It is based on the scoring method by Scholz *et al.* [20] who validated the scores by histology. They showed that almost 98% of keel bones with score 4 did not have callus material, about half of keel bones scored 3 had callus material as did the vast majority of keel bones scored 2 and 1. The occurrence of multiple fractures within one bird was not differentiated from single fractures. Therefore, the number of birds possibly acquiring a new fracture excluded hens with already palpated fractures.

At 43 weeks of age, the color marked hens were weighed and the presence of bumblefoot was determined (score 1 of Tauson *et al.* [21]). At 64 weeks of age, all birds were weighed and the presence of bumblefoot was again determined. Each scoring event and weighing took place during the dark phase when almost all hens were perching. They were gently lifted from the perch and investigated. Afterwards, they were placed on the perch again. The last assessment of the keel bone took place before depopulation. All animals were videotaped during the light period on three consecutive days every four weeks. Palpations were conducted on the first and the last day of the recordings. Hens that had acquired a fracture during the video days were subsequently observed on video to determine if a fall occurred.

### 2.3. Data Analysis

The period from the age of 19 weeks onwards was partitioned into 11.5 laying periods of 28 days each. The number of eggs per hen was estimated by the registrations in the nest. In laying period 1, only stays in the nest of 20 min. and longer starting within 3.5 h after light-on were counted as egg laying. From laying period 2 onwards, nest registrations during the first 5 h after light-on were counted since all eggs were laid during that time. Therefore, stays in the nest were categorized whether they occurred during the first 5 h or later in the day. To check the reliability of the hen registrations, the difference between the number of eggs in each nest and the estimated number (assuming 1 egg/hen/day) were tested against the hypothesis that the difference was 0 using a *t*-Test, treating pens as independent units.

To determine the onset of keel bone damage, the results of palpations were sorted by age for each bird. The first time scores reflecting damage (*i.e.*, 1, 2, or 3) were recorded and interpreted as damage having occurred.

For analysis of egg laying, nonlinear models were fitted with SAS Proc NLIN (SAS^®^ 9.2, Cary, NC, USA). The Gompertz model was:

$$y\left(t\right)=asymptote\times e^{e\left(b-c\times age\right)}$$

with the three parameters asymptote, *b*—constant, *c*—growth rate.

The logistic model was taken from Nelder in Savegnago *et al.* [22]:

$$y\left(t\right)=asymptote\left[1+e^{\left(-ct\right)}\right]-e^{-xt}$$

with the four parameters: asymptote, *b*—constant, *i*—peak growth rate and *x*—decrease after peak. Egg production was fitted using a logistic model [22].

To compare the fit of the models, the second order of Akaike’s information criterion and the coefficient of determination (*r*^2^) were used [23].

The relationship between the first egg and the final keel bone score for each individual hen was modeled by the ordered multinomial model with a cumulative logit link by Proc GENMOD (SAS^®^, Cary, NC, USA) using pen as the subject factor. Analyses involving total egg counts were performed without the hens that died during the study.

The rate of new deviations was determined among the hens without any damage. For each palpation date (*N* = 43), the number of new deviations was divided by the number of hens without any keel bone deviation or fracture before this date. Due to the distribution of this variable, three categories were made: 0, less than 0.0105 new deviations per hen, and more than 0.0105 new deviations per hen. A survival model (Proc LIFETEST, SAS^®^, Cary, NC, USA) was used to test differences of incurring a fracture among white and brown hens.

Data and residuals were checked for homogeneity of variances and for normality with the Shapiro-Wilk test. When data were normally distributed, a Pearson correlation coefficient (*r*_p_) was calculated; otherwise we used Spearman’s correlation coefficient (*r*_s_). The durations in the nest during egg laying on the 10 days before and after the first record of a keel bone fracture for each hen were logarithmically transformed and a mixed linear model was used (Proc MIXED, SAS^®^, Cary, NC, USA). The hybrid and whether the date was before or after the occurrence of the fracture were modeled as fixed effects. The pen was taken as a random effect and hen nested in pen was taken as the subject variable. Controlling for age effects, the same analyses were repeated by keeping the point of time constant for all hens regardless whether they had a fracture. The chosen day was the second day of the fifth laying period, which was the median day of the occurrence of fractures. Similarly, egg laying rates before and after fracture were compared by subtracting the rate during the 28 days after the first appearance of a keel bone fracture from the rate in the period before. As an additional control, the same was done for individuals without fractures until the end of the trial by taking the difference between rates during 28 days before and after the median day of first occurrence of fractures of all birds. The differences for both groups were additionally compared in a general linear model that considered group, hybrid, and their interaction as fixed effects (Proc GLM, SAS^®^, Cary, NC, USA). In this model, individuals with fractures occurring during laying periods 1 and 2 were excluded because laying rates were highly variable at the start of egg laying.

## 3. Results and Discussion

### 3.1. Keel Bone Deviations

**Figure 1:**
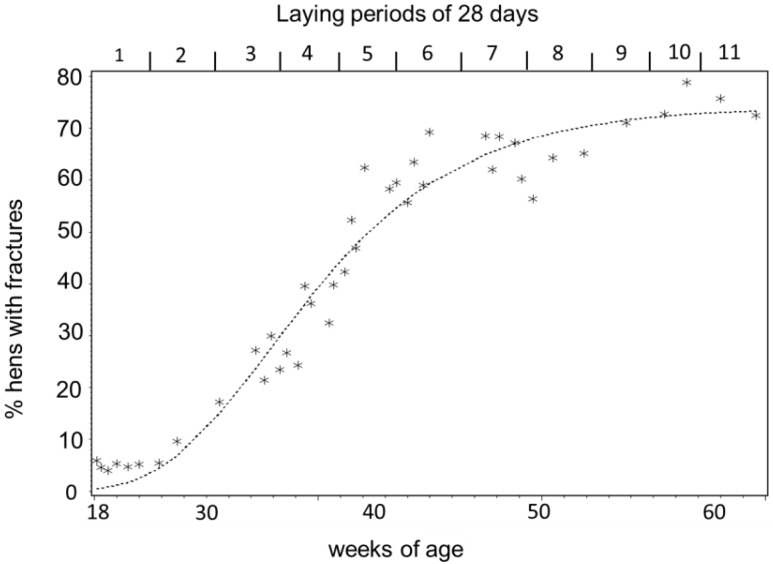
Percentage of hens with fractures according to their age (*N* = 80) and their laying periods. The time in the laying house starting at 18 weeks of age was divided into 11.5 laying periods of 28 days each. The dotted line denotes the fitted Gompertz curve.

**Figure 2:**
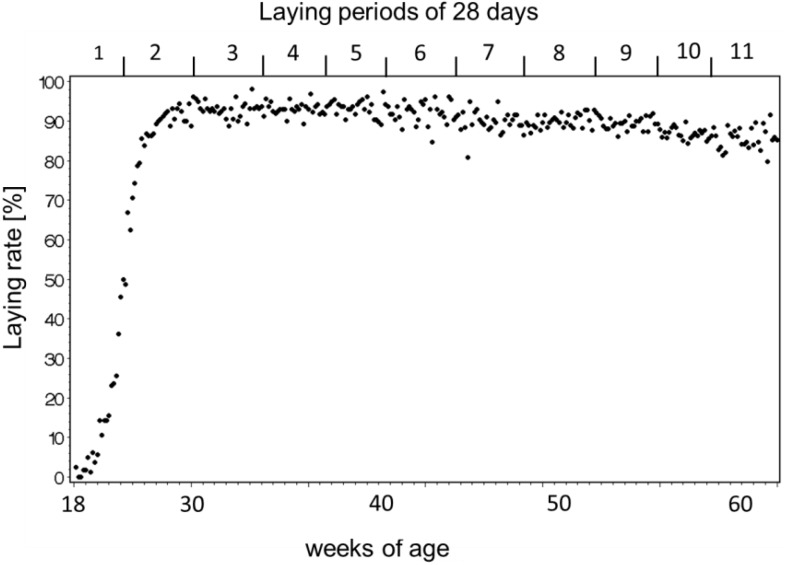
Laying rate (number of eggs per live hens in percent) during production.

**Figure 3:**
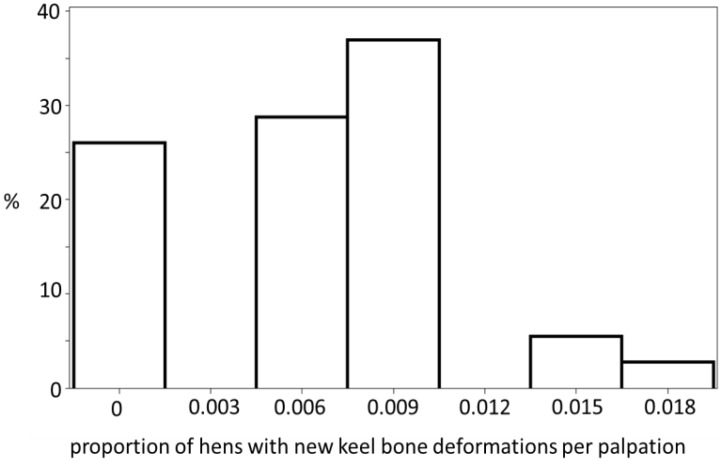
Histogram of the rate of new deviations during the 43 palpations during the laying cycle. This means that in about 26% of palpation events no (midpoint), new keel bone deviations/fractures per palpation and hen (of the hens without any deviations or fractures before that time) were detected, in 30% of palpation events, six of 1000 hens without any deviations or fractures before that time (midpoint) per palpation had a new deviation/fracture, *etc*.

The number of hens with keel bone deviations manifested a non-linear curve with increasing age. The data manifested a low prevalence during laying period 1 before the start of egg laying, a phase with rapid accumulation at the peak of lay during laying periods 2 to 6, and a phase with fewer new deviations after the peak of lay after laying period 7 (Figure 1 and Figure 2). The best fit for the increase of hens with deviations with aging was provided by a Gompertz curve (AIC_corrected_ and *R*^2^: Gompertz 145.13, 0.99, logistic 146.06, 0.99, linear 188.18, 0.87, *N* = 42). The parameters were 74.1% ± 2.5% (asymptote), 0.0210 ± 0.002 (growth rate), and 1.6466 ± 0.2 (constant). The fitted parameters for egg production were 96.5% ± 3.7% (asymptote), 20.03 ± 1.70 (days after first egg when egg production reached its peak), 0.16 ± 0.004 (constant), and 3.8 × 10−4 ± 2.0 × 10−5 (rate of production decrease after the peak). The peak of egg production was reached at the end of laying period 2. In laying period 7 and beyond, laying rate dropped below 90% for at least two days in a row. Therefore three production phases were defined: Pre-peak (laying period 1), peak laying (laying periods 2 to 6), and post-peak (laying periods 7 to 11). The frequencies of new deviations fell into three categories: 0, ≤0.0105 deviations per hen, and >0.0105 per hen and date (Figure 3). New deviations occurred more often during peak production than before or after the peak (Table 1, χ^2^_4_ = 12.58, Fisher’s exact test: *p* < 0.0055, *N* = 43).

**Table 1:** The frequencies of new fractures fell into three categories of 0, up to 0.0105 deviations per hen, and more than 0.0105 per hen (see Figure 3) per date of palpation. In total, 43 palpations were performed.

Time	No New Deviations	<0.0105	>0.0105
Laying period 1	4	2	0
Periods 2–6	5	17	2
Periods 7–11.5	10	3	0

**Figure 4:**
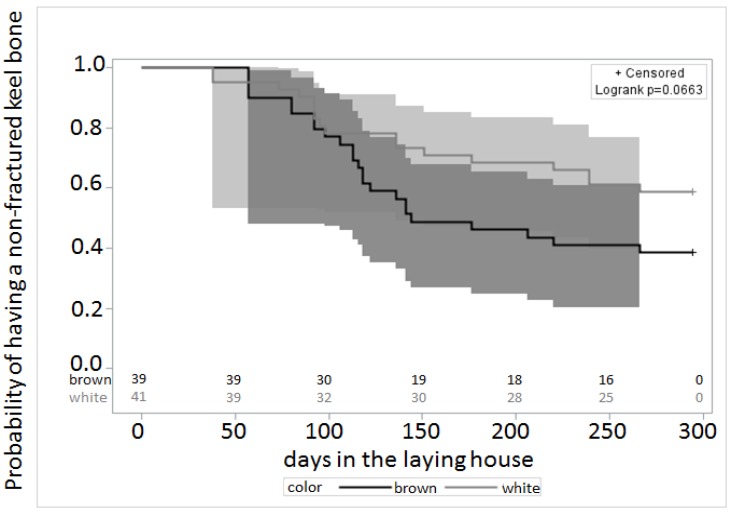
Probabilities of having a non-fractured keel bone during the course of the study as obtained from the survival analysis. The dark grey (for brown hens) and the light grey (for white hens) show the 95% confidence intervals of the survival curves.

There was a trend for brown hens to develop more keel bone fractures (Figure 4). The mortality between hens with and without fractures did not differ (χ^2^_1_ = 0.0025, *p* = 1).

### 3.2. Palpation

Scores between two consecutive palpations changed in 19.7% of the cases. The change from score 4 to score 3 was the most frequent followed by the change from 3 to 4 (Table 2). The different changes occurred at different ages (Kruskal Wallis Test: χ^2^_5_ = 75.40, *p* < 0.0001). Changes from score 4 to score 3 were earlier than changes from 4 to 2 (χ^2^_1_ = 5.38, *p* = 0.02). The number of changes were not affected by the time span between palpations (Spearman rank correlation, *r*_s_ = −0.19, *p* = 0.58, *N* = 11).

**Table 2:** The frequency of changes of palpation scores from one palpation to the next.

Change	Number	% of Total	% of Changes	Median Age (Weeks)
Period change	2532	80.03	-	
4 to 3	161	5.1	26.0	36
4 to 2	34	1.08	5.48	38.3
4 to 1	3	0.09	0.48	47.9
From 3 to 2	118	3.74	19.03	41
From 3 to 1	0	0	0	-
From 2 to 1	32	3.74	5.16	42.9
From 1 to 2	27	0.86	4.36	46.4
From 1 to 3	0	0	0	-
From 1 to 4	0	0	0	-
From 2 to 3	100	3.17	16.13	41.1
From 2 to 4	11	0.35	1.77	41
From 3 to 4	42	1.08	6.77	37

### 3.3. Egg-Laying and Keel Bone Score

The number of estimated eggs and counted eggs in the nests did not differ significantly (*t* = 1.23, *p* = 0.25, *N* = 8 pens).

**Figure 5:**
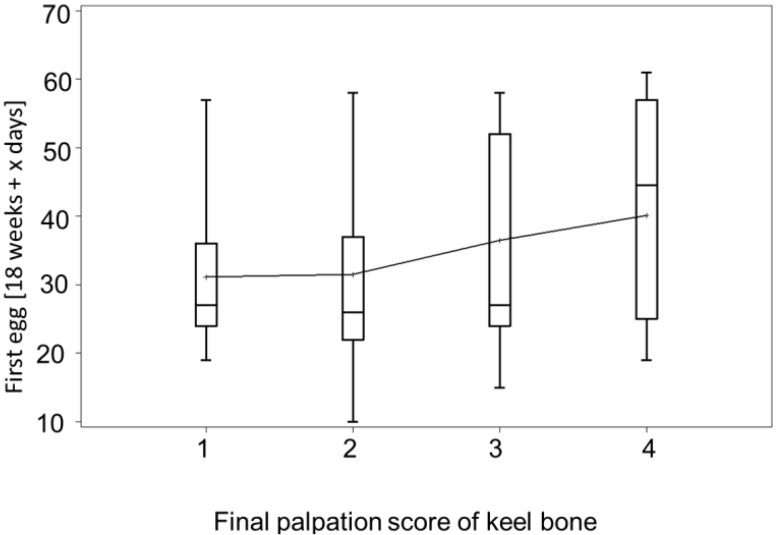
Boxplot of the age when the first egg was laid for hens with different palpation scores at the end of the laying period. Means are connected for better visibility. Hens with better (=higher) score of the keel bone had laid their first egg at a later age than hens with lower scores.

An earlier appearance of a hen’s first egg was associated with a worse keel score at final palpation (begin: χ^2^_1_ = 4.23, *p* = 0.04, hybrid: χ^2^_1_ = 0.25, *p* = 0.62, *N* = 121) (Figure 5). However, the total number of eggs was not associated with the final score (χ^2^_1_ = 0.04, *p* = 0.849, *N* = 103). There was a trend for a negative correlation between the age at first egg and the total number of eggs (*r*_p_ = −0.188, *p* = 0.058, *N* = 103).

There was no difference in the rate of egg laying during the 28 days before and after keel bone fracture (mean difference ± S_Error_ = 0.04 ± 0.025 eggs/day, *t* = 1.47, *p* = 0.15, *N* = 34). To account for age-related changes, this group of hens was compared with control hens without fractures (see Methods). There was a marginally significant interaction between group (hens with and without fractures) and hybrid (white and brown) for the difference in laying rates (GLM, group: *F* = 1.39, 1 df, *p* = 0.24, hybrid: *F* = 0.28, 1 df, *p* = 0.60, group*hybrid: *F* = 4.08, 1 df, *p* = 0.048). This would suggest that the laying rates differed in hens with and without fractures. However, the interaction was due to a (non-significant following *post hoc* testing) difference in laying rates in white hens before and after fracture (least square means of difference: 0.08 for white hens with fracture, −0.02 for white hens without fracture) so the effect of fracture on the change in laying rates was weak at best.

### 3.4. Bumblefoot

At 43 weeks of age, 12.7% of hens (10 hens) had one and 6.3% (five hens) had two bumblefeet. Bumblefoot occurred in both hybrids and there was no difference between them (Fisher’s exact test *p* > 0.74). The occurrence with bumblefoot was associated with the presence of fractures at the end of the laying period (χ^2^_6_ = 17.73, Fisher’s exact test: *p* = 0.01, *N* = 80, Table 3). At the end of the laying period when all hens were assessed, 6.9% had one bumblefoot (8 hens), none had two. Eleven hens had improved and only one hen had a bumblefoot at the end of the laying period, which had had healthy feet at 43 weeks of age. Three hens retained the bumblefoot condition. There was no increased mortality associated with the occurrence of bumblefoot at the age of 43 weeks months (one hen with bumblefoot and six hens without bumblefoot died between 43 weeks of age and the end of the production period).

**Table 3:** Bumble feet were associated with deformed (score 3) and fractured (scores 1 and 2) keel bones at 65 weeks of age. For a description of scores, see Methods.

Bumble Foot	Keel Bone			
	Score 1	Score 2	Score 3	Score 4
No bumble foot	2	23	18	21
One bumble foot	2	2	1	5
Two bumble feet	0	6	0	0
Total	4	31	19	26

χ^2^_6_ = 17.73, Fisher’s exact test: *p* = 0.01, *N* = 80.

### 3.5. Behavior

**Figure 6:**
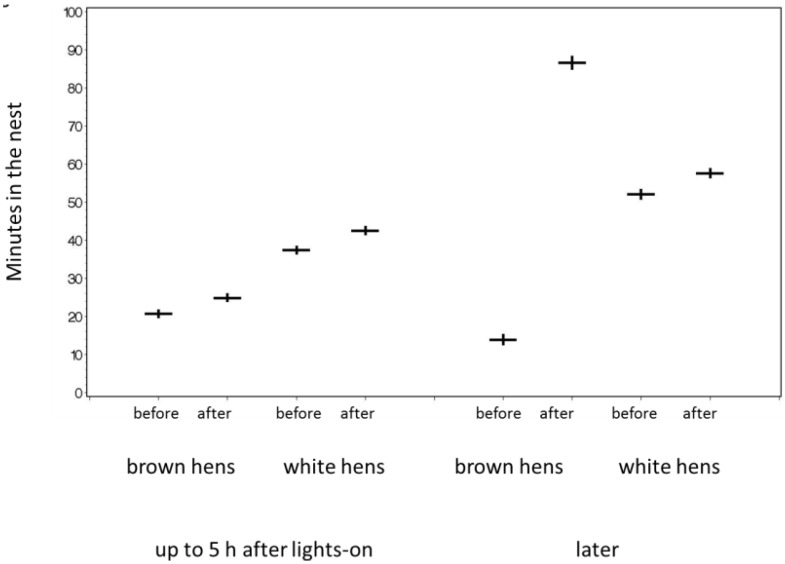
Hens were longer in the nest during the 10 days after the fracture of the keel bone than during the 10 days before the fracture (back transformed least square means). Means (horizontal bars) and standard errors (vertical bars) are given.

No flight accidents were observed during recorded video that could be linked to the instance of fracturing the keel bone. Except for a few individuals all hens perched at night regardless of the fracture status. However, hens with new fractures spent more time in the nest during 10 days after fracture compared to the 10 days before the fracture (with scores 3 or 4). This was the case during the first 5 h after light-on (*F*_1,633_ = 6.73, *p* = 0.01, *N* = 41 hens) and later (*F*_1,53_ = 5.29, *p* = 0.026, *N* = 18) (Figure 6). Taking the median day of occurrence for all birds instead of the days of fracture for the particular birds found no significant effects between the 10-day period before and after that day except between hybrids (within 5 h after light-on: before/after: *F*_1,1843_ = 0, *p* = 0.95, *N* = 129, hybrid: *F*_1,1843_ = 59.96, *p* < 0.0001, *N* = 129; afterwards: before/after: *F*_1,138_ = 0.21, *NS*, *N* = 42, hybrid: *F*_1,138_ = 1.17, *NS*, *N* = 42).

### 3.6. Discussion

To our knowledge, this is the first longitudinal study correlating the occurrence of keel bone deviations and egg laying over a full production cycle of laying hens. For about half of the hens, the approximate date of acquiring a fracture was known and at least 61.6% had a fracture at the end of production. We base the interpretation of our scoring system on the histological examinations of Scholz *et al*. [20] who found callus material indicating fractures in almost all hens with keel bone scores 1 and 2 and part of 3. The frequency of fractures is in line with reports from other flocks of the same farm [19,24] as well as other farms and countries [1,5,25]. Palpation is a subjective measurement and part of the changes in palpation scores in Table 2 might be attributed to misdiagnosis [18]. The same keel bone might be scored 3 or 4 at different times when deviations were minor. Despite some fluctuations in palpation scores, there were consistent patterns. More severe fractures happened later than less severe deviations and fractures. A new fracture score 2 was more frequently found in keel bones that had previously been scored as deformed (=score 3) than intact (=score 4). This supports the validity of the palpation scores.

At the moment, not much is known about the processes during bone healing to make precise statements on the timing of fracture and bone structure. If there is (just) a crack, this might not be obvious during palpation until callus material forms and the fracture might be detected sometimes after the incident. It is possible that callus material enlarges for some time so that the same fracture is first scored as 3 and later, with a bigger callus, as 2. However, keel bones where bone pieces were displaced could be scored as a score 2 or 1 before the callus formation had occurred. This scoring was likely in a rather short period after the keel bone got severely fractured. The assumption that a fracture occurs directly after the impact is not proven, either. Sometimes, a force acting on material can lead to tensions that result in a fracture at a later time without an impact. In other words, the first appearance of score 2 or less (called “fracture”) was the best approximation when the fracture happened but could indicate a date after the fracture.

The occurrence of fractures was temporally linked to egg laying where most fractures occurred at the peak of egg production and not when most accidents were expected to occur while housed in a new barn [24,26]. A link with egg laying was also shown by the negative correlation between the age of the first egg and the probability of having a fracture at the end of production. Interestingly, there was no relationship between the total number of eggs and fractures or between the total number of eggs and the age at first egg. One reason might be that egg production during early periods is not genetically correlated with total egg production [27]. Whitehead [28] suggested that the length of the egg laying period influences the probability of fracture.

Two studies have found a negative influence of keel bone fractures on egg production [11,29], however we did not find a significant difference in egg laying rates before and after fractures of individual hens. There are various possible reasons for this discrepancy. Possibly, hens with weaker bones prone to break lay fewer eggs some time before the occurrence of fracture. Two phenomena might interfere: Hens with high rates of egg laying might be more prone to fractures but their laying rates might drop sometime after the occurrence of fracture. Due to the high variation in egg laying capacity, severity of damage, and time of the damage occurring, sample size might have been too small in the current study to detect significant differences. A possible indication for lower egg production after keel bone fracture could be lower feed intake [30], but we did not measure feed intake. An additional source of variation is that the time when most fractures occur is also the period when laying rates naturally decrease with age making it harder to detect a difference between birds with intact and broken keel bones.

Apart from physiological factors related to calcium metabolism falls and collisions play a role for keel bone damage as Stratmann *et al.* [26] showed. Hens with bumblefoot might have been more likely to lose grip on the perch and fall. After picking up the hens, it took longer to place hens with bumblefoot on the perch again than hens with healthy feet (own obs.).

The increase of time spent in the nest during egg laying in birds with fractures might be due to pain during laying while hens delay the act of laying or difficulties of laying due to changed shell properties of the egg. A change of egg shell characteristics of hens with fractures has also been found [29]. There are indications for both possibilities, because hens with keel bone fractures move less from perch to ground than hens with intact keel bones or those with fractures in the presence of analgesics [11,12,31]. Some hens with newly acquired fractures might have been hiding in the nests during the day to avoid locomotion or interaction with other hens. Occupancy of a nest by two hens was possible and was seen on video. However, hens seen entering an occupied nest left this nest quite quickly after entering. The way the RFID antennas were constructed (one antenna pad consisted of 12 small antennas), all hens on the antenna pad were registered at the same time and short occupancies were not counted. Disturbances by other hens entering the nests should not be related to keel bone status of the hen in the nest so it would increase the error variance of nest occupancy. Interestingly, almost all hens perched at night regardless of the condition of their keel bone. Perching at night might be a behavior without phenotypic plasticity because of strong natural selection for escaping predation in wild chickens.

Differences between hybrids were small, but there was a trend for brown hens to develop more fractures. It is known that brown hens spend less time in the nest as was shown here [32], but there was no significant interaction between hybrid and the effect of keel bone fracture.

We did not find an association between keel bone fractures and mortality. The most common cause of mortality was bleeding due to toe injuries, infections of the oviduct and unknown causes.

## 4. Conclusions

In conclusion, although strict causation cannot be inferred with any observational study, a causal relationship between keel bone fractures and the timing of egg laying seems likely. An environmental impact through accidents causing keel bone damage could not be demonstrated. More detailed automated registration methods of movements of individual birds might be necessary to monitor accidents leading to fractures. A behavioral change in egg laying behavior after keel bone fractures and birds with new fractures hiding in nest boxes supports the notion that keel bone fractures could be painful for the birds.
